# Management of Peritoneal Dialysis-Associated Emergencies during the COVID-19 Pandemic: The Experience of a Center of Excellence

**DOI:** 10.3390/life14070805

**Published:** 2024-06-26

**Authors:** Cristian Iorga, Cristina Raluca Iorga, Iuliana Andreiana, Simona Hildegard Stancu, Iustinian Bengulescu, Traian Constantin, Victor Strambu

**Affiliations:** 1 Faculty of Medicine, “Carol Davila” University of Medicine and Pharmacy, 050474 Bucharest, Romania; cris.iorga@yahoo.com (C.I.); simonastancu2003@yahoo.com (S.H.S.); iustinian.bengulescu@umfcd.ro (I.B.); traianc29@yahoo.com (T.C.); drstrambu@gmail.com (V.S.); 2 Surgery Clinic, “Dr. Carol Davila” Clinical Nephrology Hospital, 010731 Bucharest, Romania; 3 Nephrology Clinic, “Dr. Carol Davila” Clinical Nephrology Hospital, 010731 Bucharest, Romania; 4 Department of Urology, “Prof. Dr. Th. Burghele” Hospital, 050652 Bucharest, Romania

**Keywords:** peritoneal dialysis emergencies, COVID pandemic, telemonitoring

## Abstract

The COVID-19 pandemic struck unexpectedly; emergency services and chronic care institutions, including dialysis centers, were overloaded. A significant problem was the care of COVID-positive patients alongside the care of chronically dialyzed patients who presented emergencies. In our hospital, which became a COVID support center for dialysis patients with severe forms of the disease, we had to care for PD patients with dialysis-related emergencies. We present two cases of patients managed on an outpatient basis or 1-day hospitalization who were treated successfully without compromising the quality of the care provided. We used remote monitoring, worked in a multidisciplinary team, and shortened the duration of the patients’ hospitalization (and implicitly the risk of contact). In pandemic conditions, the advantage of PD was the possibility of patient isolation; in the first 6 months of the pandemic, we recorded no deaths in this category of patients. In hemodialysis patients, infection and mortality rates were high. Although we expected an increase in the number of peritoneal dialysis patients in the post-pandemic period, this did not happen. We continue to plead for the popularization of the PD method among patients and doctors, which has proven advantages in pandemic conditions.

## 1. Introduction

The respiratory disease caused by the new coronavirus, declared a pandemic by the WHO on 11 March 2020, overturned medical systems all over the world, including in Romania [1]. 

Romania was at an advantage because its pandemic started later (the first case was confirmed on 26 February), giving our country the necessary time to prepare and be inspired by the experience of the previously severely affected countries.

In 1974, the first iterative hemodialysis center in the country was established at the “Dr. Carol Davila” Nephrology Clinical Hospital, which has continued uninterrupted activity to this day.

The modernization of the hospital has been an ongoing process, and today, it has 215 beds, a nephrology department, a surgical department (including vascular surgery), allergology, urology, and an intensive care unit.

In essence, our facility is a comprehensive centralized center for the care of renal patients, among others.

In 2020, by the order of the Minister of Health, our hospital was declared a support hospital for dialysis cases seriously affected by COVID-19. 

As a consequence, the organizational structure of the hospital was changed, and separate functional circuits for patients, staff, and patients infected with COVID had to be created [2].

The major advantage of our facility is its pavilion-style architectural structure with two separate buildings, making the complete separation of facilities for patients infected with COVID-19 a possibility.

At the time, under the conditions of the restrictions placed on current medical personnel and medical activity, we had to face a new challenge—the uninterrupted care of peritoneal dialysis patients so that they could continue to benefit from dialysis as well as the management of possible associated emergencies [3,4,5,6].

## 2. Materials and Methods

According to recommendations, a telemonitoring system was implemented so that hospital visits were transformed into audio–video conferences using platforms for remote communication (WhatsApp, Zoom, and smartphones) [7,8,9,10].

Following the experience of other centers, we limited the access of peritoneal dialysis patients to the hospital.

They were instead contacted every 3 days by medical staff who ensured that they had the necessary supplies at home; they checked on their state of health and offered advice for at-home care.

The hospital’s dialysis center directly cares for 42 hemodialysis patients and 13 peritoneal dialysis patients, but the real total number of dialysis patients who are dependent on “Dr Carol Davila” Hospital is a few hundred a year, serving practically the entire southern area of the country.

However, even under these conditions, emergency situations arose that required hospitalization.

The results of the implementation of the new protocols were verified by evaluating the first two cases that required emergency patient care.

Our previous experience from the successful collaboration of the surgical team with a team of nephrologists and the intensive care unit (ICU) led to these results.

## 3. Results

### 3.1. Case 1

A 71-year-old male patient has been under our care since October 2018, at that time with dialysis-dependent acute kidney injury, not recovered. 

As the patient was diabetic and presented significant cardiovascular morbidity (cerebral lacunar disease, ischemic cardiomyopathy with chronic heart failure (CHF) NYHA class III, peripheral arteriopathy of the lower limbs stage IV, and arterial fibrillation permanently on anticoagulant treatment), our team proposed peritoneal dialysis as chronic renal replacement therapy, an option that the patient and his wife accepted. 

A straight PD Tenckhoff catheter was implanted in the left flank, with continuous ambulatory peritoneal dialysis (CAPD) with four exchanges daily, cleaning and dressing of the exit site every other day, and local antibiotic therapy with mupirocin and intranasal application of mupirocin, 5 days/month (as the patient is a nasal carrier of Staphylococcus aureus).

The clinical and biological evaluation was favorable.

At the beginning of March 2020, the patient presented with erythema, purulent secretion, and pain at the catheter exit site.

**Figure 1:**
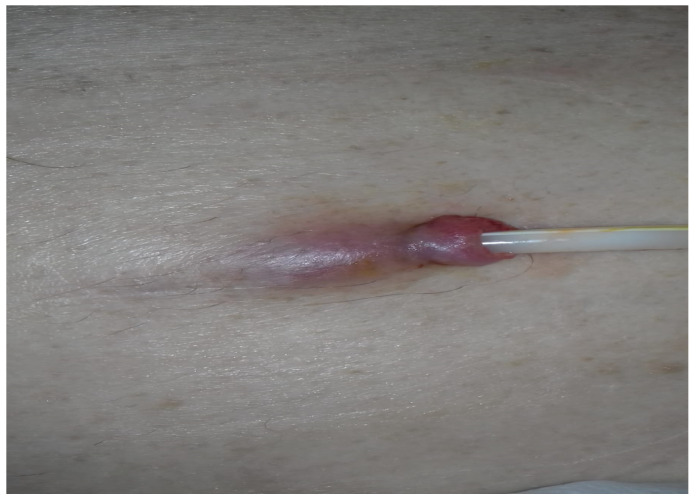
Tumefaction and erythema of the exit site and the initial part of the subcutaneous tunnel.

He was diagnosed with exit-site infection associated with tunnel infection (Figure 1).

We initiated empiric antibiotic therapy with oral levofloxacin 250 mg/day and rifampicin 600 mg/day and intensified the local treatment (washing and disinfection with hydrogen peroxide and hypertonic saline solution and antibiotic therapy with kanamycin ointment).

Since there was no improvement after a week, we augmented the treatment with vancomycin 2 g intraperitoneally every 5 days, without response at 3 weeks. We decided to extract the catheter and to proceed with contralateral implantation at the same operative time, with the aim of minimizing the length of hospitalization.

We talked to the patient’s family, and his wife agreed to take responsibility for wound dressing and home peritoneal dialysis treatment according to our instructions.

Following a telephone consultation, a therapeutic plan for the patient was established, being scheduled for hospital admission on 4 July 2020. 

The medical recommendations were as follows:

- to interrupt oral anticoagulant and antiplatelet administration one week prior to admission, prevention being ensured by in-house administration of low-molecular-weight heparin;
- to monitor temperature twice a day;
- to monitor the appearance of the peritoneal fluid;
- to monitor the wound from the catheter exteriorization opening;
- to maintain complete isolation at home throughout the period, without outside contact.

The hospitalization was scheduled so that both the extraction of the infected catheter and the implantation of a new peritoneal dialysis catheter in the contralateral flank could be performed in the same surgical session.

The pre-anesthesia consultation was initially carried out remotely.

Taking into account the patient’s medical history, local anesthesia and intravenous sedation were recommended (we avoided general anesthesia with orotracheal intubation, which carried increased risks for both the patient and medical staff).

On the day of admission, biological samples were collected, and anesthesia and surgical consultations were performed.

After obtaining the results, the patient was transported to the operating room, where staff were equipped with increased means of protection.

During the same surgical session, the infected catheter was extracted (the surgical wound of the exit point was left open) and the insertion of a new dialysis catheter in the right flank was performed without incident via the open method.

The patient was discharged the next day, being transported home by ambulance. Postoperatively, he required daily telephone monitoring by the surgeon and the nephrologist.

We recommended a stringent restricted diet of fluids, fresh fruits and vegetables due to his minimal residual renal function (diuresis 500 mL, eGFR 4 mL/min).

The washing of the peritoneal cavity was performed once every two days. Wound dressing was performed daily.

The culture results taken intraoperatively from the tip of the catheter revealed the presence of Staphylococcus aureus, sensitive to clarithromycin, gentamicin, linezolid, and vancomycin, which justified the continuation of antibiotic therapy with vancomycin administered intraperitoneally in a liter of 1.5% glucose solution overnight, every 5 days, for two more weeks postoperatively.

From the 5th postoperative day, we progressively restarted the peritoneal dialysis treatment, initially of two exchanges/day with 1 liter of dialysis solution each time, with the patient in supine position. 

From the 8th postoperative day, we increased the dialysis dose by adding two more volume exchanges of 500 mL. 

The volumes were gradually increased every two days until the patient reached the stable schedule he had prior to the surgery.

The evolution was favorable, both from a surgical (primary healing of wounds without signs of local complications) and nephrological (early resumption of peritoneal exchanges and clear appearance of liquid without leakage at the suture points) point of view.

### 3.2. The Particularities of The Case

The patient had multiple associated co-morbidities, which required preoperative preparation and postoperative care. Our advantages were that the patient was already present in the records of the clinic and his medical conditions were already known; therefore, treatment and care recommendations could be easily transmitted and applied.

The patient was dependent at home on care provided by another person (his wife).

### 3.3. Case 2

A 63-year-old male patient presented himself at the emergency room on 4 May 2020, requesting consultation for diffuse abdominal pain and dialysis effluent turbidity in the last 48 h.

The patient was not under the care of our nephrology center, so all information about previous medical history was provided by the patient himself.

Renal replacement therapy by peritoneal dialysis was initiated in 2019.

About 2 weeks prior to this visit to our clinic, he observed pus purulent secretion at the catheter exit site for which he was successfully treated with levofloxacin.

Upon presentation, the patient was in good general condition, without fever. He complained of spontaneous and on palpation diffuse abdominal pain which did not require administration of analgesics and showed no signs of peritoneal irritation; the intestinal transit was delayed, the peritoneal effluent appeared cloudy, and ultrafiltration was negative (−200 mL) for the last two days. Systemic inflammation and high effluent cellularity were present (reactive protein C 230 mg/L, 6 800 cells/mm^3^, 92% PMN).

A diagnosis of peritoneal dialysis-associated peritonitis (first episode) was established. 

The patient remained under observation for about 6 h in our emergency department. We carried out one exchange of peritoneal fluid with antibiotics (2 g vancomycin), and afterwards the patient went home. It was his own wish (he refused hospitalization), and the protocol of the hospital at that time had restrictions on patients’ admission. 

Bacteriology samples were collected from the exit site and from the dialysis effluent. Empiric antibiotic therapy with levofloxacin 250 mg/day orally and vancomycin 2 g intraperitoneally every 5 days was initiated. The culture showed Staphylococcus aureus at the exit site and in the intraperitoneal fluid.

We recommended that he monitor his temperature twice daily (or as needed), monitor the peritoneal fluid and the exit site of the catheter, and call us or his attending nephrologist if abdominal pain, fever, or purulent discharge occurred. 

The patient was consulted in our emergency department because he could not contact his attending physician; after that, he was monitored by telephone daily by the attending nephrologist.

His evolution was favorable, with the clearing of dialysis liquid after 2 days of treatment.

The total treatment duration was three weeks.

### 3.4. The Particularities of The Case

This patient had recently been started on peritoneal dialysis (approximately 6 months prior) and presented with a severe complication—peritonitis. The patient was not known to our clinic, but after following the treatment instructions received, the evolution of the case was uneventful and without the appearance of other complications requiring hospitalization/presentation to the hospital.

Under normal circumstances, the patient would have been hospitalized, but in the context of the spread of SARS-CoV-2, we decided on treatment and follow-up at home.

The two cases emphasize one of the advantages of peritoneal dialysis, namely independence from hospital management and, in special conditions, the possibility of treating complications.

## 4. Discussion

The SARS-CoV-2 pandemic has of now caused up to 704 million infections in the world, of which over 7 million resulted in death.

In Romania, on 11 May 2020, 15,362 positive cases and 952 deaths were registered, only 2 and half months after the start of the COVID-19 pandemic [2].

Similar to situations in other countries, more than half of contaminations occurred in closed communities—nursing homes for the elderly, institutions for disabled people, hemodialysis centers, and centers for palliative care. 

In these places, the number of infections increased exponentially, with more than half of the deaths in the country being represented by people in these communities [4,11,12,13].

In particular, hemodialysis patients are at high risk of contracting the disease, and mortality is increased in this category [14,15,16].

Comparatively, in the private hemodialysis centers, there were mass outbreaks of illness with COVID-19 involving both patients and medical and auxiliary personnel, with 100 deaths recorded among hemodialysis patients infected with SARS-CoV-2 in the 6 months after the start of the pandemic [17].

Regarding the deaths caused by the SARS-CoV-2 pandemic in our country, no deaths of peritoneal dialysis patients were recorded (compared to approximately 100 deaths among hemodialyzed patients in the first 6 months). This is another justification for increasing the number of peritoneally dialyzed patients, not only in Romania but also in the wider world [15,18,19,20].

In 2022, in our country, there were 18,253 dialysis patients, of which 326 were treated by peritoneal dialysis [20].

Although Romania is a success story related to peritoneal dialysis, just as in all the countries of the world, upon the introduction of private dialysis centers, the number of peritoneal dialysis patients decreased steadily compared to the number of hemodialysis patients.

In 2012, a prediction from the RRR (Romanian Renal Registry) showed that had the trend been maintained, in 5 years, Romania would have reached a 60% decrease in the number of peritoneal dialysis patients; however, in 2020, Romania had the same number of PD patients [20]. However, the data from the RRR in 2020 show a 60% increase in new patients on peritoneal dialysis. The year 2021 continued this trend, with a decrease of 44% in new patients in peritoneal dialysis, followed by an increase of 18% in 2022 [20]. The main issue of the decrease in patients on peritoneal dialysis is low reimbursement.

Another explanation of the low number of PD patients is the experience of nephrologists. In Romania, we have around five departments dedicated to PD patients’ care. This means we have a lack of information, a lack of education, and a lack of prescriptions to PD patients. 

However, the pandemic years have shown more advantages of this dialysis method, and this is a reason that more and more patients trust this method.

Studies prior to the COVID-19 pandemic tried to demonstrate the utility of remote control of peritoneal dialysis patients, as confirmed by Milan Manai et al. and later by Virizi et al. Studies have also indicated that remote control increases patients’ trust in this dialysis modality [21,22,23].

In 2024, the WHO indicated the tremendous importance of digital technologies that are already part of people’s life for the scope of health and wellbeing [24].

PD is the best example of the use of remote control and telemedicine in the care of patients. However, it requires patient education, electronic devices, and good internet connection. It also needs a coherent legislative context and reimbursement for the medical act.

Unfortunately, we did not gain any more patients being treated with PD, but the number was similar to that before pandemic. This indicates that is not necessarily a matter of patient trust, but a matter of education and information. This is another issue that e-Health could address.

## 5. Conclusions

Therapeutic success in conditions of unfavorable medical support is equally due to the multidisciplinary team involved in the treatment of the dialysis patients and the patients’ close adherence to treatment along with family support.

Each type of dialysis has advantages and disadvantages, indications, and contraindications, but the reality of the recent pandemic has emphasized a new advantage of PD both in Romania and worldwide—it allowed for social distancing and remote follow-up of patients so that no deaths were recorded among these patients in the first 6 months of the SARS-CoV-2 pandemic.

## Data Availability

Data are contained within the article.
